# Transcriptome response of Atlantic salmon (*Salmo salar*) to competition with ecologically similar non‐native species

**DOI:** 10.1002/ece3.3798

**Published:** 2018-01-08

**Authors:** Xiaoping He, Aimee Lee S. Houde, Bryan D. Neff, Daniel D. Heath

**Affiliations:** ^1^ Great Lakes Institute for Environmental Research University of Windsor Windsor ON Canada; ^2^ Department of Biology Western University London ON Canada; ^3^ Present address: Pacific Biological Station, Fisheries and Oceans Canada Nanaimo BC Canada

**Keywords:** gene expression, interspecific competition, non‐native species, reintroduction, RNA‐Seq, salmonids

## Abstract

Non‐native species may be introduced either intentionally or unintentionally, and their impact can range from benign to highly disruptive. Non‐native salmonids were introduced into Lake Ontario, Canada, to provide recreational fishing opportunities; however, the establishment of those species has been proposed as a significant barrier to the reintroduction of native Atlantic salmon (*Salmo salar*) due to intense interspecific competition. In this study, we compared population differences of Atlantic salmon in transcriptome response to interspecific competition. We reared Atlantic salmon from two populations (LaHave River and Sebago Lake) with fish of each of three non‐native salmonids (Chinook salmon *Oncorhynchus tshawytscha*, rainbow trout *O. mykiss,* and brown trout *S. trutta*) in artificial streams. We used RNA‐seq to assess transcriptome differences between the Atlantic salmon populations and the responses of these populations to the interspecific competition treatments after 10 months of competition in the stream tanks. We found that population differences in gene expression were generally greater than the effects of interspecific competition. Interestingly, we found that the two Atlantic salmon populations exhibited similar responses to interspecific competition based on functional gene ontologies, but the specific genes within those ontologies were different. Our transcriptome analyses suggest that the most stressful competitor (as measured by the highest number of differentially expressed genes) differs between the two study populations. Our transcriptome characterization highlights the importance of source population selection for conservation applications, as organisms with different evolutionary histories can possess different transcriptional responses to the same biotic stressors. The results also indicate that generalized predictions of the response of native species to interactions with introduced species may not be appropriate without incorporating potential population‐specific response to introduced species.

## 1 INTRODUCTION

The wide establishment of non‐native species is one of the major global environmental challenges caused by human activities. It is estimated that there are close to a half million exotic species introduced into different ecosystems worldwide (Pimentel et al., 2001). On one hand, introduced species can provide conservation values to local ecosystems. For example, non‐native plants can provide habitat for native species (Severns & Warren, 2008; Sogge, Sferra, & Paxton, 2008), and non‐native animals (e.g., crayfish and round goby) can be food sources for threatened native species resulting in increased abundance for these native species (King, Ray, & Stanford, 2006; Tablado, Tella, Sánchez‐Zapata, & Hiraldo, 2010). On the other hand, introduced species more often threaten local biodiversity through pathogen introduction and increased predation and competition (Manchester & Bullock, 2000; McDowall, 2003; Peeler, Oidtmann, Midtlyng, Miossec, & Gozlan, 2010; Vitule, Freire, & Simberloff, 2009).

Notably, there is increasing evidence that the presence of introduced species can affect the growth, reproduction, and survival of ecologically similar native species because of interspecific competition (Houde, Smith, Wilson, Peres‐Neto, & Neff, 2016; Houde, Wilson, & Neff, 2015a; Scott, Noakes, Beamish, & Carl, 2003). Interspecific competition affects lower social status species disproportionately (Gilmour, DiBattista, & Thomas, 2005), and the effects of interspecific competition are similar to those associated with a chronic stress response, that is, decreased growth, loss of immune function, and reduced survival (Barton, 2002; Gilmour et al., 2005). Therefore, the presence of non‐native species can be considered a chronic stressor for native species, potentially causing declines in abundance of threatened native species as well as being a significant barrier to the reintroduction of locally extirpated native species.

The presence of established non‐native salmonids in Lake Ontario and its tributaries has been proposed as a significant barrier to the successful reintroduction of Atlantic salmon (*Salmo salar*) (Jones & Stanfield, 1993; Scott, Poos, Noakes, & Beamish, 2005). Atlantic salmon was a native species in Lake Ontario until extirpated in the late 1800s, and decades of reintroduction efforts have been largely unsuccessful (Dimond & Smitka, 2005). A number of non‐native salmonid species (Chinook salmon *Oncorhynchus tshawytscha*, coho salmon *O. kisutch*, rainbow trout *O. mykiss,* and brown trout *S. trutta*) have been successfully introduced into Lake Ontario to address the recreational demand for large salmonid sport fishes (Stewart & Schaner, 2002). Some of the established non‐native species, such as rainbow trout and brown trout, tend to be more aggressive than Atlantic salmon (Van Zwol, Neff, & Wilson, 2012), and these species can be considered biotic stressors for Atlantic salmon. Although it is generally difficult to demonstrate the effects of interspecific competition in natural systems (Hastings, 1987), experiments using artificial systems are a good alternative because physical environmental variables can be largely controlled. Studies using artificial streams revealed that competition with juvenile rainbow trout and brown trout had negative effects on the growth and survival of juvenile Atlantic salmon (Houde, Wilson, & Neff, 2015b; Houde et al., 2015a). The effects of interspecific competition in natural stream sites are similar to those demonstrated in artificial habitats, as observed for Atlantic salmon with rainbow trout (Houde et al., 2016). Furthermore, although juvenile Chinook salmon were found to have no negative effects on juvenile Atlantic salmon growth or survival (Houde et al., 2015a), adult Chinook salmon can affect the survival of adult Atlantic salmon during reproduction (Scott et al., 2003). Those studies demonstrated that interspecific competition between Atlantic salmon and non‐native salmonids can affect the establishment of Atlantic salmon after release into Lake Ontario, but the mechanisms that mediate the negative effects on Atlantic salmon at the molecular level are unknown.

Gene expression is the process whereby genetic information stored in the genome is used to synthesize functional products that determine phenotype. Gene expression is influenced by both genetic and environmental factors (Buckland, 2004; Hodgins‐Davis & Townsend, 2009; López‐Maury, Marguerat, & Bähler, 2008; Petretto et al., 2006). In particular, changes in gene expression are the mechanisms associated with acclimation and adaption to environmental stressors (Schulte, 2004). Although gene expression changes in response to abiotic stressors have deepened our understanding of population differences in response to, and tolerance of factors such as, thermal stress (Narum & Campbell, 2015), pollution exposure (Whitehead, Triant, Champlin, & Nacci, 2010), and salinity (Brennan, Galvez, & Whitehead, 2015), most transcriptional studies using biotic stressors have focused on responses to immune challenges (e.g., Wellband & Heath, 2013), and differences among individuals of different social rank (e.g., Schunter, Vollmer, Macpherson, & Pascual, 2014; Trainor & Hofmann, 2007). To our knowledge, studies of the transcriptional responses to interspecific competition (and differences among populations) have not been reported for any organism.

In this study, we reared Atlantic salmon with each of three ecologically similar non‐native salmonids (Chinook salmon, rainbow trout, and brown trout) which are established in Lake Ontario. We used two Atlantic salmon populations (LaHave and Sebago) to examine transcriptional responses to interspecific competition. These two populations are being used for reintroduction into Lake Ontario because they have been successfully introduced into other lakes (Dimond & Smitka, 2005). Our goal was to compare population differences in transcriptome response of Atlantic salmon to interspecific competition with non‐native salmonids, to characterize the molecular genetic mechanisms underlying differential tolerance of biotic stress caused by interspecific competition. We collected samples after 10 months of common rearing (competition) in artificial streams with four treatments per population: Atlantic salmon reared alone and Atlantic salmon reared with each of the three non‐native salmonids. We used RNA‐Seq to compare the transcriptome response of the two Atlantic salmon populations to the interspecific competition treatments.

## 2 METHODS

### 2.1 Design and sampling

Two Atlantic salmon populations were used in this study: LaHave River and Sebago Lake. For each Atlantic salmon population, we created four treatments: Atlantic salmon reared alone and Atlantic salmon reared with one of three salmonids (Chinook salmon, rainbow trout, and brown trout). All fish used in this study were provided by the Ontario Ministry of Natural Resources and Forestry (OMNRF). Fish were produced from fertilizations in fall 2011 (Atlantic salmon, Chinook salmon, and brown trout) and spring 2012 (rainbow trout). Atlantic salmon eggs were reared at OMNRF Codrington Research Facility, and fry of the other three species were transferred to Codrington in spring of 2012. Fish from the four salmonid species were transferred to artificial stream tanks in September 2012, and the interspecific competition experiment was conducted until July 2013. Initially, there were 32 Atlantic salmon in each of the tanks where Atlantic salmon were reared alone, and there were 16 Atlantic salmon and 16 fish of the competing species in each of the tanks where Atlantic salmon were reared with the non‐native species. After 10 months in the artificial stream tanks, Atlantic salmon were humanely euthanized using an overdose of buffered MS‐222. We collected spleens from the juvenile Atlantic salmon and stored them in RNAlater. We chose to sample spleen tissue for this study because the spleen is sensitive to whole‐organism chronic stress and is associated with circulating red blood cells and immune response (Hernandez et al., 2013; Peters & Schwarzer, 1985).

### 2.2 RNA isolation

RNA was extracted from spleen tissue using Trizol (Invitrogen, CA, USA) following the manufacturer's instructions. The quality and concentration of RNA were checked using Agilent RNA 6000 Nano Kit in an Agilent 2100 Bioanalyzer (Agilent Technologies, Mississauga, ON, Canada). We selected RNA samples with RNA integrity number (RIN) >7.0 from four Atlantic salmon individuals within the same competition treatment, and these samples were pooled using equal amounts of total RNA. The pooled RNA samples were treated for possible genomic DNA contamination using TURBO^™^ DNase (Invitrogen). After DNase treatment, the quality and concentration of the pooled RNA samples were checked again using the Agilent 2100 Bioanalyzer (Agilent Technologies) and sent to BGI Americas Corporation for RNA sequencing. We pooled samples of four fish to minimize the effect of individual transcriptional variation and focus on the general influence of interspecific competition on the transcriptome of Atlantic salmon. Although pooling eliminates our ability to assess interindividual variation, it is cost‐effective and maximizes the design for testing for competition effects. For Atlantic salmon reared alone, we sent two separate pooled RNA samples for each population. For Atlantic salmon reared with each of the other three species, we sent one pooled RNA sample for each population. In total, 10 pooled RNA samples were sent for RNA sequencing performed on the Illumina HiSeq^™^ 2000 platform.

### 2.3 Data analysis

To obtain sequence read counts for each gene, we followed the protocol described in Anders et al. (2013). Briefly, paired and cleaned reads (i.e., Phred quality score > 20) were mapped to the Atlantic salmon genome (NCBI accession no.: AGKD00000000.3) using Bowtie 1.1.1 (Langmead, Trapnell, Pop, & Salzberg, 2009) and Tophat 2.0.13 (Trapnell et al., 2012) using default parameters. Then, we used samtools 1.2 (Li et al., 2009) to sort and create the SAM files. After that, we used HTSeq‐0.6.1 (Anders, Pyl, & Huber, 2015) to count reads for each gene with parameters “–stranded=no” and “–a = 10.” We used Cufflinks to obtain the mRNA sequences of each gene (Trapnell et al., 2012). The longest isoform of each gene was extracted and used for blastx search in the nonredundant protein database using blast 2.2.30 with a cutoff e‐value of 1e^−5^ (Camacho et al., 2009). The results obtained from local blastx were loaded into Blast2GO (Conesa et al., 2005) for GO term mapping and annotation.

Transcriptional data were examined using unsupervised and supervised approaches. For the unsupervised approach, we used principle component analyses and constructed distance heatmaps using DESeq2 (Love, Huber, & Anders, 2014). For the supervised approach, we tested for transcriptomic response to competition with the three different non‐native salmonid species within each Atlantic salmon population using GFOLD V1.1.3 (Feng et al., 2012). GFOLD can analyze RNA‐Seq data with biological replicates or one treatment without replicates within a reliable statistical framework (Feng et al., 2012). To prepare gene expression data for GFOLD, we followed GFOLD's recommendations to obtain reads per kilobase of transcript per million mapped reads (RPKM) value for each gene in each sample. To identify differentially expressed genes by GFOLD, c value was set to 0.01 as default and genes with GFOLD value larger than 1 or less than −1 were accepted as significantly differently expressed. To quantify transcriptomic response to interspecific competition, we compared the Atlantic salmon reared with one non‐native species to the two control samples (Atlantic salmon alone) within each population. The functional categorization of significantly differentially expressed genes in response to interspecific competition was plotted using BGI WEGO (Ye et al., 2006).

### 2.4 Quantitative real‐time PCR

To validate RNA‐Seq results, we designed qRT‐PCR primers for 14 genes (Table S1) which showed significant differences in at least one of the six comparisons among four pooled samples: LaAS1 (LaHave Atlantic salmon reared alone sample 1), LaBT (LaHave Atlantic salmon reared with brown trout), SeAS1 (Sebago Atlantic salmon reared alone sample 1), and SeBT (Sebago Atlantic salmon reared with brown trout) as identified by GFOLD. We used *ribosomal protein S20* (*rps20*) as the endogenous control as it has been shown to be invariant in Atlantic salmon spleen (Olsvik, Lie, Jordal, Nilsen, & Hordvik, 2005). We measured gene expression for three individuals from each of the four pooled samples in four treatments: LaHave reared alone, LaHave reared with brown trout, Sebago reared alone, and Sebago reared with brown trout. For each individual, we had three technical replicates. TURBO^™^ DNase‐treated RNA was used for cDNA synthesis using the High‐Capacity cDNA Reverse Transcription Kit (Applied Biosystems, Burlington, ON, Canada). The cDNA samples were diluted 1:10 for qRT‐PCR analysis. The qRT‐PCR reactions were conducted in 10 μl reactions which consisted of 5 μl SYBR Select Master Mix (Applied Biosystems), 0.5 μl 10 μmol/l primers, and 1 μl diluted cDNA. The qRT‐PCR was performed in a QuantStudio 12K Flex Real‐Time PCR System (Applied Biosystems). The relative expression of each targeted gene was normalized to the expression of *rps20*.

## 3 RESULTS

### 3.1 Sequencing summary and reads mapping

The RNA‐Seq data have been submitted to the NCBI SRA database (SRA accession: SRP080309). In total, we obtained approximately 320 million 90 bp high‐quality (Q > 20) reads (160 million left reads and 160 million right reads) from the 10 pooled RNA samples. The number of sequences per sample ranged from 29.8 million to 33.9 million (Table S2). Overall, 78.8% of the reads mapped to the Atlantic salmon draft genome and the mapping rate for each sample varied from 72.9% to 81.3%. In total, 92,812 transcripts were recovered, and the average size of transcripts was 2,038 ± 1,710 bp (Mean ± *SD*) (ranged from 91 to 15,642 bp).

### 3.2 Principle component analysis and distance heatmap

**Figure 1:**
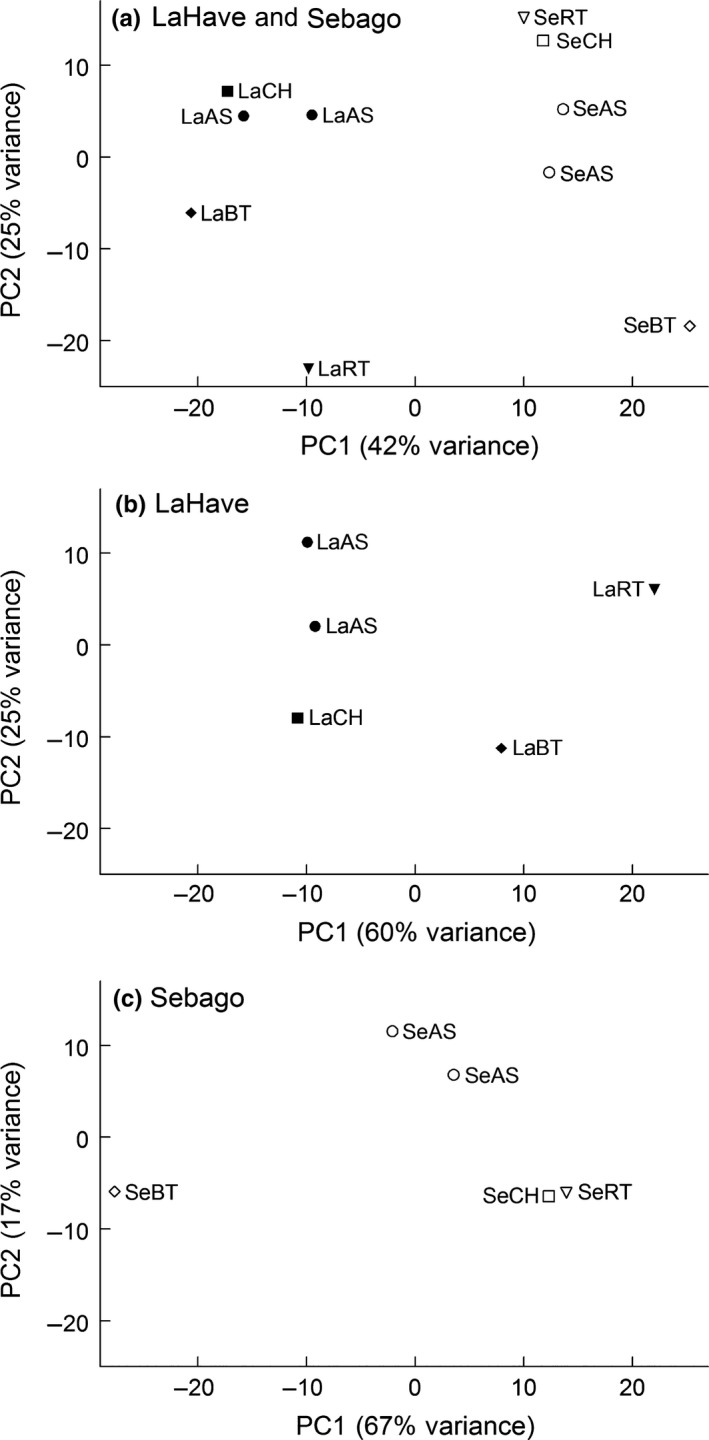
Principal component analysis based on transcription in juvenile Atlantic salmon for 2,000 selected genes which exhibited the highest expression variation among samples for (a) all the 10 samples, (b) the LaHave population samples (*n* = 5) and (c) the Sebago population samples (*n* = 5). Treatment symbols: LaAS indicates LaHave Atlantic salmon reared alone; LaBT, LaCH, and LaRT indicate LaHave Atlantic salmon reared with one of the three species: brown trout, Chinook salmon, and rainbow trout, respectively; SeAS indicates Sebago Atlantic salmon reared alone; SeBT, SeCH, and SeRT indicate Sebago Atlantic salmon reared with one of the three species: brown trout, Chinook salmon, and rainbow trout, respectively

Principle component analysis based on gene transcription of the 2,000 genes which had the highest variation among samples showed that PC1 and PC2 explained 42% and 25% of the variance, respectively (Figure 1a). The two populations were separated by PC1, while PC2 primarily reflected variation among competition treatments. However, clear population‐specific responses to competition were evident. For example, competition with rainbow trout resulted in the largest transcriptional response in the LaHave population, while competition with brown trout resulted in the largest transcriptional response in the Sebago population.

Within each population, Atlantic salmon responded differently to competition with the three introduced salmonid species (Figures 1b and c). The LaHave Atlantic salmon reared with Chinook salmon showed a different transcriptome response compared to the LaHave Atlantic salmon reared with rainbow trout along both PC1 and PC2 (Figure 1b). The Sebago Atlantic salmon reared with Chinook salmon showed a similar transcriptome response to the Sebago Atlantic salmon reared with rainbow trout, and the Sebago Atlantic salmon under these two treatments showed different transcriptome response compared to the Sebago reared with brown trout (Figure 1c).

**Figure 2:**
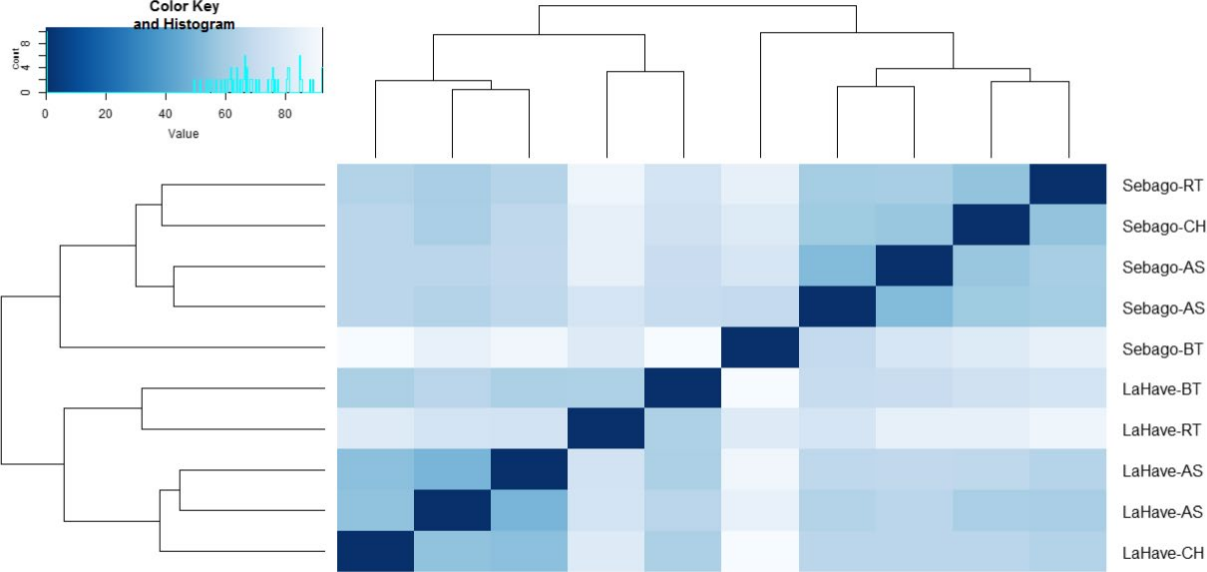
Heatmap of sample‐to‐sample distances based on transcription in juvenile Atlantic salmon from two populations (LaHave and Sebago) for all genes scored. Treatment symbols: AS indicates Atlantic salmon reared alone; BT, CH, and RT indicate the Atlantic salmon population reared with one of the three species: brown trout, Chinook salmon, and rainbow trout, respectively

In the distance heatmap, the five samples within each population clustered together, reflecting the large population effect on the transcriptome (Figure 2). Within the LaHave population, the Atlantic salmon reared alone and Chinook salmon competition samples clustered together, while the LaHave Atlantic salmon reared with rainbow trout and with brown trout clustered together. Within the Sebago population, the two Atlantic salmon reared alone samples clustered together, while the Atlantic salmon reared with Chinook salmon and with rainbow trout clustered together. The Atlantic salmon reared with brown trout showed a highly divergent transcriptional profile within the Sebago population. The distance heatmap and PCA analyses indicated that the effects of population on gene expression were greater than those of interspecific competition.

### 3.3 Gene expression differences

**Figure 3:**
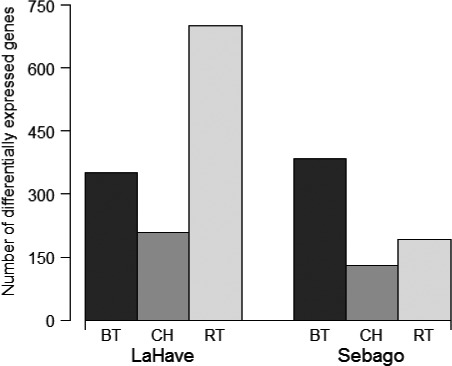
Number of genes that exhibited a significant transcriptional response in juvenile Atlantic salmon (*Salmo salar*) to the presence of brown trout (BT), Chinook salmon (CH), and rainbow trout (RT). Atlantic salmon derived from two populations (LaHave and Sebago)

Within the LaHave population, there were 209, 350, and 701 genes that exhibited a significant response to competition with Chinook salmon, brown trout, and rainbow trout, respectively (Figure 3). Within the Sebago population, there were 131, 384, and 191 genes that responded to competition with Chinook salmon, brown trout, and rainbow trout, respectively (Figure 3). Within LaHave, there were 10 genes that exhibited a significant response to all three interspecific competition treatments (Figure S1a; Table S3). Within Sebago, there were nine genes that responded significantly to all three interspecific competition treatments (Figure S1b; Table S4).

There were only 23, 13, and 19 genes shared between the two populations in response to competition with Chinook salmon, brown trout, and rainbow trout, respectively. Among the 23 genes showing a common response to competition with Chinook salmon in both populations, 20 showed the same trend of regulation of gene expression (Table S5). Among the 13 genes showing a common response to competition with brown trout in both populations, three showed the same trend of regulation (Table S6). Among the 19 genes showing a common response to competition with rainbow trout in both populations, six showed the same trend of regulation (Table S7). While most responding genes were population‐specific, the GO term analysis using the combined responding genes within each population showed that the responding genes were involved in similar functional groups in the two study populations (Figure S2).

### 3.4 Comparison between gene expression level revealed by qRT‐PCR and RNA‐seq

We quantified transcription at 14 genes in 12 fish from four treatments (LaHave reared alone, LaHave reared with brown trout, Sebago reared alone, and Sebago reared with brown trout) using qRT‐PCR. The Spearman correlation coefficient between relative expression quantified by qRT‐PCR and RNA‐Seq was 0.81 (Figure S3).

## 4 DISCUSSION

The establishment of non‐native species can negatively affect the fitness of less aggressive native species (Fausch, 2007; Turek, Pegg, & Pope, 2013). While gene expression response to many environmental stressors has been reported (Brennan et al., 2015; Narum & Campbell, 2015; Wellband & Heath, 2013; Whitehead et al., 2010), transcriptional responses to competition with ecologically similar species have not been reported in any vertebrates to our knowledge. In this study, we used RNA‐Seq to compare transcriptome responses of two Atlantic salmon populations to competition with ecological similar species with known dominance ranks. Overall, the effects of population on gene transcription were higher than those of interspecific competition, and there were both differences (responding genes) and similarities (functional groups of responding genes) between Atlantic salmon populations in response to competition with ecologically similar species at the gene expression level.

Previous studies found that competition with rainbow trout or brown trout can have negative effects on the growth and survival of Atlantic salmon, while competition with Chinook salmon had no negative effects (Houde et al., 2015a; Van Zwol et al., 2012). In this study, we found that Atlantic salmon had fewer genes responding to competition with Chinook salmon than to competition with rainbow trout or brown trout. The larger transcriptional responses to rainbow trout and brown trout were expected because these species tend to be more aggressive than Atlantic salmon, while Atlantic salmon tend to be as aggressive as Chinook salmon (Houde et al., 2015a; Van Zwol et al., 2012). We also found that the number of responding genes for Atlantic salmon in the presence of brown trout was similar between populations (350 in LaHave and 384 in Sebago, respectively), suggesting that the presence of brown trout affects both Atlantic salmon populations similarly at the transcriptome level. However, the two Atlantic salmon populations showed substantial differences in the number of genes responding to competition with rainbow trout. That is, the number of genes responding to the presence of rainbow trout in the LaHave population was 3.6 times the number in the Sebago population. This suggests that the Sebago population may be more tolerant to the presence of rainbow trout than the LaHave population, and thus, the Sebago population may be more suitable for reintroduction in Lake Ontario as rainbow trout are common in many of the tributaries of the lake (Stanfield, Gibson, & Borwick, 2006).

Among competition treatments within each population, LaHave Atlantic salmon reared with rainbow trout had the highest number of genes showing a significant response, while Sebago Atlantic salmon reared with brown trout had the most responding genes. Unlike previous results for the effects of interspecific competition on fitness‐related traits which concluded that brown trout is the most serious competitor to Atlantic salmon and that rainbow trout can also have negative effects (Houde et al., 2015a; Van Zwol et al., 2012), our results indicate that the most stressful competitor to the LaHave Atlantic salmon may be rainbow trout, while the most stressful competitor to the Sebago Atlantic salmon may be brown trout, at least in the surviving Atlantic salmon in this study. Thus, transcriptomic tools may be more sensitive to interspecies competition effects than commonly used fitness‐related phenotypic traits.

Shared responding genes in the two populations likely reflect conserved transcriptional responses and may thus be used as candidate genes in other systems for interspecific competition studies. In response to competition with Chinook salmon, five genes encoding apolipoproteins consistently showed downregulation in both populations. Apolipoproteins play an important role in lipid metabolism via lipid transport (Li, Tanimura, Luo, Datta, & Chan, 1988). In response to competition with rainbow trout, two *somatostatin* genes and the *glucagon 1* gene showed downregulation in both populations. Somatostatin is a hormone that participates in multiple biological processes and can inhibit the release of pituitary hormones and gastrointestinal tract peptides (Burgus, Ling, Butcher, & Guillemin, 1973; Gahete et al., 2010). Glucagon plays an important role in glucose homeostasis by regulating blood glucose concentration (Quesada, Tudurí, Ripoll, & Nadal, 2008). Our results show that interspecific competition downregulates metabolic genes of Atlantic salmon which are related to lipid and glucose metabolism. Although chronic stress is thought to decrease immunity and disease resistance in fish (Barton, 2002), we found very few genes related to immune response with significant transcriptional response to interspecific competition, indicating that chronic stress caused by interspecific competition likely does not affect the immune system of fish.

Conserved transcriptomic response to interspecific competition is also reflected at the gene functional categorization of the responding genes. Although the overlap in the specific genes responding in the two populations is low, the similarity in GO terms of differentially expressed genes indicates that they may have evolved different gene networks to achieve the same results. This also highlights the need for a transcriptome approach to study population differences in stress response as populations can use different pathways to achieve the same outcome.

Populations can have stress response differences due to their different evolutionary histories (He, Johansson, & Heath, 2016), and those differences at the gene expression level can deepen our understanding of stress tolerance differences. Within the Sebago population, six genes consistently showed downregulation in competition with three non‐native salmonids. Among the six genes, three are *somatostatin* genes. Somatostatin has been reported to regulate social behavior in cichlid fish (*Astatotilapia burtoni*) (Trainor & Hofmann, 2006), with dominant males having larger somatostatin‐containing neurons and higher expression of the *somatostatin* and *somatostatin receptor 3* genes in the hypothalamus relative to subdominant males (Hofmann & Fernald, 2000; Trainor & Hofmann, 2007). Additionally, Schunter et al. (2014) found the *somatostatin receptor 1* gene showed higher expression in the brain of territorial males than females in *Tripterygion delaisi* during the reproductive period. Although the functions of somatostatin genes in the spleen are not clear, the downregulation of expression of these genes in competition with rainbow trout in both populations and in competition with the three species in the Sebago population may be adaptive because of the reported negative feedback regulation roles of somatostatin (Gahete et al., 2010). Among the six genes responding to competition with the three non‐native salmonids in the Sebago population, two genes are *glucagon 1* and *insulin* both of which have opposite roles in regulating blood glucose concentration (Quesada et al., 2008). With the LaHave population, the shared genes in response to the three non‐native salmonid have no obvious role related to stress response. Thus, the specific responding genes shared within each population implied that it is likely there was more adaptive response in the Sebago population. This supports the previous results that the Sebago population likely has higher competitive ability with non‐native salmonid than the LaHave population (Houde et al., 2015a,b, 2016).

## 5 CONCLUSIONS

This study is the first report of transcriptomic responses of native species to interspecific competition with ecologically similar introduced species in a vertebrate. We found both similarities and differences in transcriptome responses to interspecific competition for the two Atlantic salmon populations. Overall, the Sebago population had fewer responding genes than the LaHave population, implying that the Sebago population was less affected by interspecific competition than the LaHave population. This study adds to the growing number of studies (Houde et al., 2015a,b, 2016; Van Zwol et al., 2012) indicating that the Sebago population likely has higher competitive ability than the LaHave population. Population similarity in competitive ability can be reflected based on functional gene ontologies, and differences can be reflected at the specific responding genes. Transcriptome characterization can be used to quantitatively and functionally evaluate differences among populations in their response to stress, such as interspecific competition, which provides objective criteria for source population selection in reintroduction efforts, among other conservation applications. More broadly, our transcriptome analyses highlight population‐specific responses to non‐native species interactions, which support the view that the impact of exotic species may depend on the ecological and environmental context of the invaded habitat (Pyšek et al., 2012).

## CONFLICT OF INTEREST

The authors declare no conflict of interest.

## AUTHOR CONTRIBUTIONS

XH collected samples, conducted laboratory work, analyzed the data, and wrote the manuscript. ALSH contributed to sample collection. ALSH and BDN took charge of fish rearing, and provided comments on the manuscript. DDH provided suggestions on data analysis and helped revise the manuscript at all stages.
