# Supplementary material for: Transcriptome response of Atlantic salmon (Salmo salar) to competition with ecologically similar non‐native species
Source: Ecol Evol. 2018 Jan 8;8(3):1769–77. doi: 10.1002/ece3.3798 (PMC5792521; doi:10.1002/ece3.3798)
Supplement: Supplementary file 1 [file ECE3-8-1769-s001.docx]

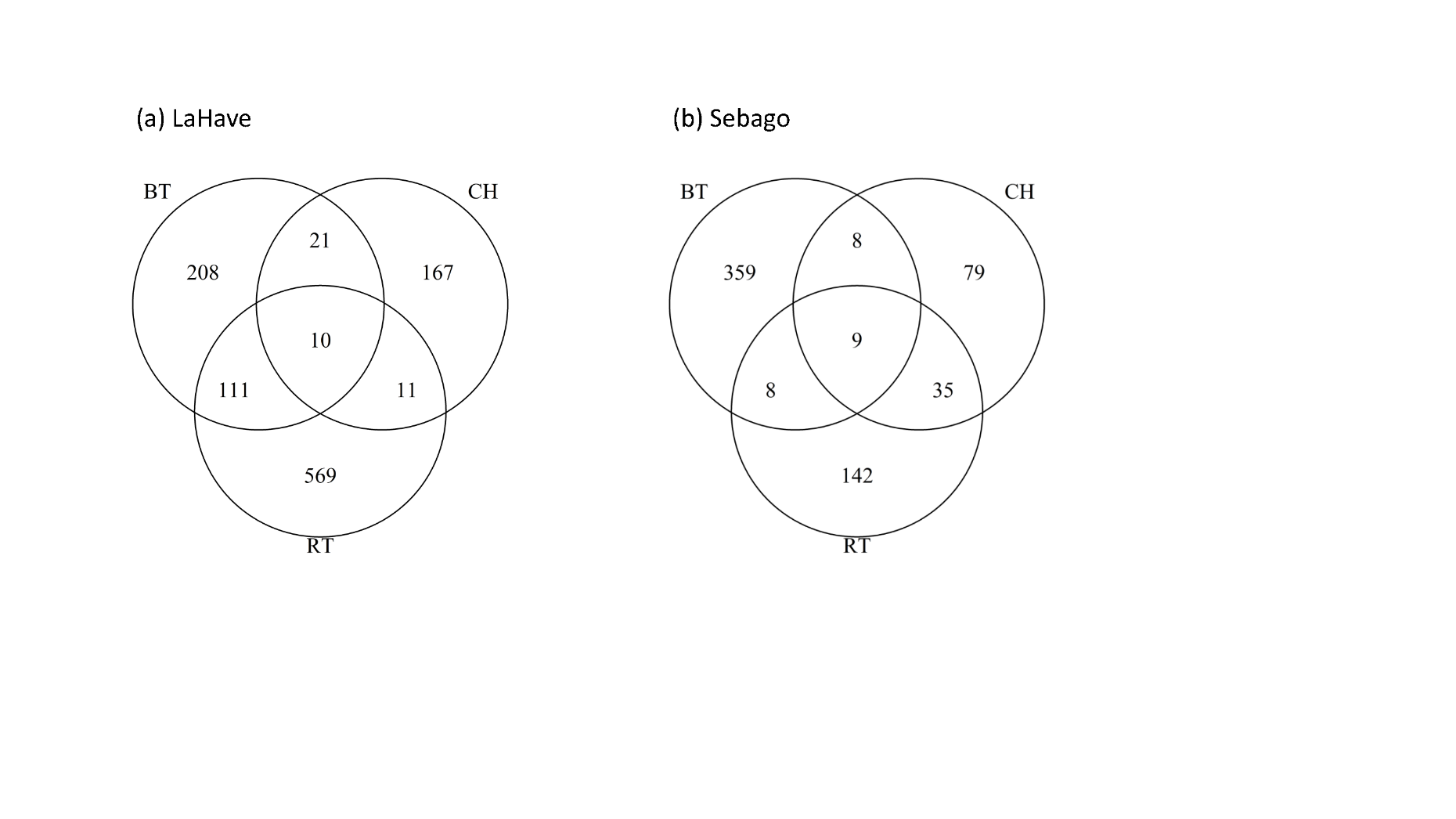


**Supplementary Figure S1** Venn diagram showing the overlap of differentially transcribed genes in response to the presence of brown trout (BT), Chinook salmon (CH) and rainbow trout (RT) in LaHave (panel a) and Sebago (panel b) juvenile Atlantic salmon.


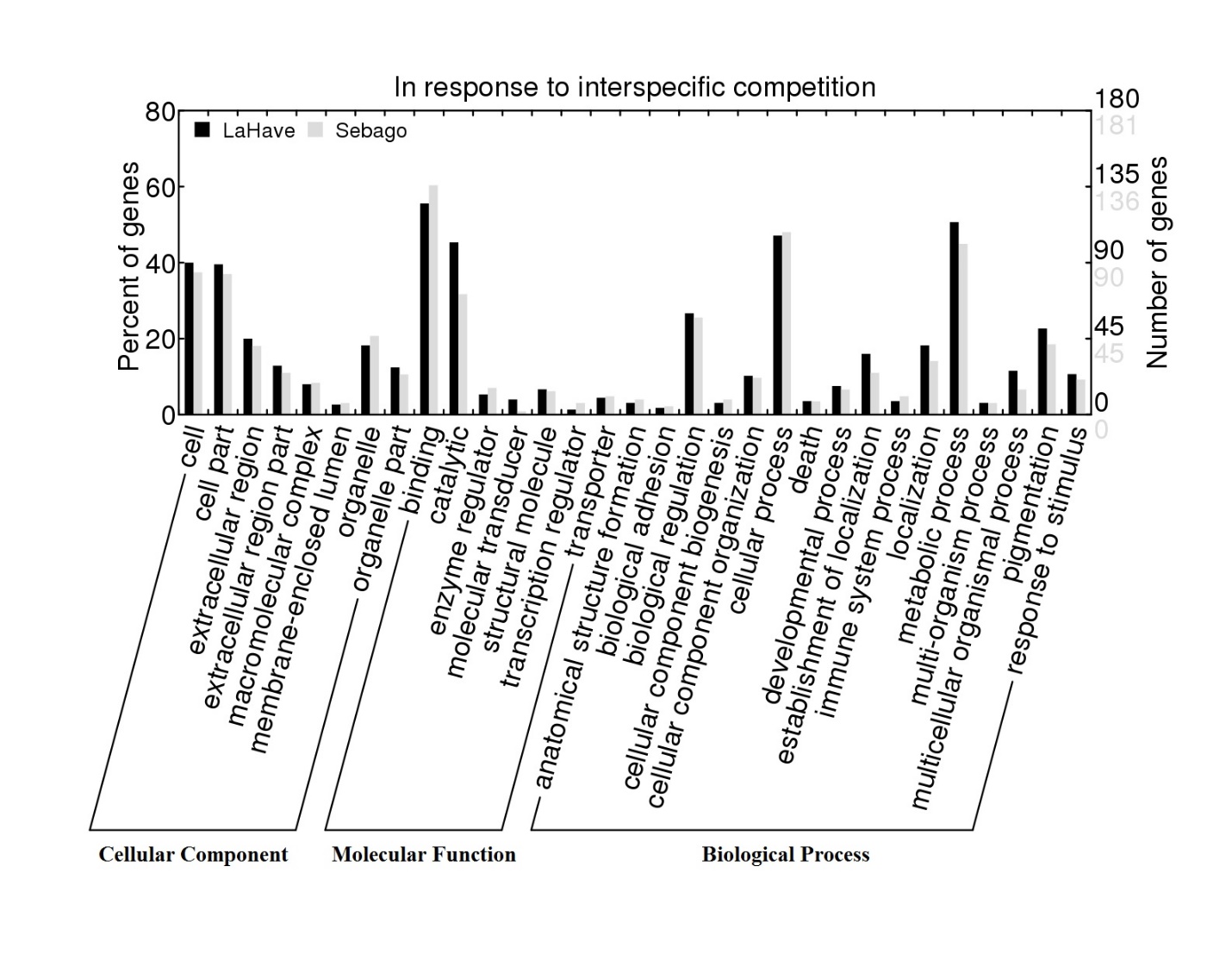


**Supplementary Figure S2** Functional categorization of significantly differentially transcribed genes in response to interspecific competition for the LaHave and Sebago juvenile Atlantic salmon (*Salmo Salar*) populations. For each population, responding genes were combined. GO terms containing less than five genes in both populations were not included.


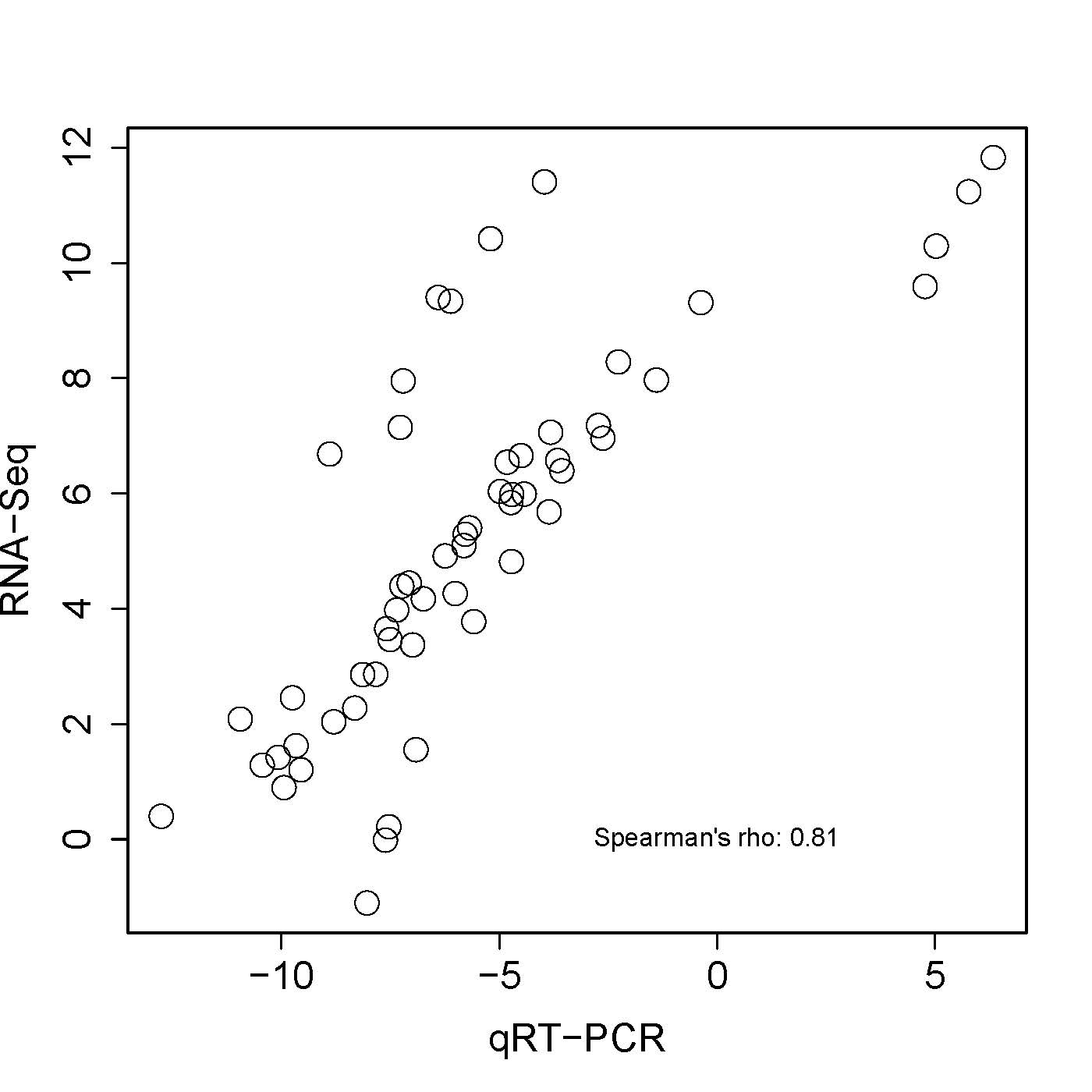


**Supplementary Figure S3** Comparison of gene transcription levels revealed by RNA-Seq and qRT-PCR for 14 genes. Expression of the 14 genes (see gene names in Table S1) for RNA-Seq were log_2_ transferred RPKM values from four samples: LaAS1 (LaHave Atlantic salmon reared alone sample 1), LaBT (LaHave Atlantic salmon reared with brown trout), SeAS1 (Sebago Atlantic salmon reared alone sample 1), and SeBT (Sebago Atlantic salmon reared with brown trout). qRT-PCR was used to quantify expression of the 14 genes for 12 individuals from the four treatments and then -ΔC_T_ (calculated by ΔC_Treference_ - ΔC_Ttargeted_) values were averaged for each treatment. Spearman rank correlation analysis was conducted using the averaged -ΔC_T_ for each treatment and log_2_RPKM.
