# Supplementary material for: Transcriptome response of Atlantic salmon (Salmo salar) to competition with ecologically similar non‐native species
Source: Ecol Evol. 2018 Jan 8;8(3):1769–77. doi: 10.1002/ece3.3798 (PMC5792521; doi:10.1002/ece3.3798)
Supplement: Supplementary file 2 [file ECE3-8-1769-s002.docx]

**Supplementary** **Table S1** Primers used for qRT-PCR to validate RNA-Seq transcriptional response in juvenile Atlantic salmon to interspecific competition.

| Gene name | Forward primer | Reverse primer |
| --- | --- | --- |
| 60 kDa lysophospholipase | GAACATACGAGGCTACGAC | TCCATCAAACAGAGGCTAA |
| Alpha amylase | CTGGCTCCCAAAGGATACGC | CTCCACGGGCTGGTCAA |
| Aquaporin-1 | ATCCTGGCTCAGATGCT | AACCCTTGTCCAACACTTATT |
| Carboxylic ester hydrolase | ACTGCCTCTACCTGAACATA | AGGGTCACCACGATTACA |
| Carboxypeptidase A1 | ATCCTGGCTCATCCTGC | CCTTGGGTCCACGGTAA |
| Carboxypeptidase A2 | TCAGGGTCAATGTGGAGTC | AGCGTACAGGCTGGAGTAG |
| Elastase-like serine protease | CCTATTGAGCCTCTGACCACC | TGTCTCCACTCACCGTCCC |
| Formin-binding protein 1 | CGACGAATAAGTAACGAGG | GATGTAGTCTATCACAGTGCC |
| Hemoglobin subunit beta-1 | CACTCCCGCAGCAATCAT | TTGTTGGCGTGGGTCTCG |
| Olfactomedin-4 | CTTTGCTGCTGATGAGAC | GGTCAATGTAACGGGTG |
| Somatostatin-1A | TGCTCCAACGGTCACTCA | AGATCCACATCCTCCTGCT |
| Splicing factor 3B subunit 4-like | GGCTATGGCTTTGTTGAGTT | CCTTGTTGACACGGATGG |
| Trypsin 1 | GCTGCTCACTGCTACAAG | AACTGCTCGCTACCCTCA |
| Trypsin 2 | TGTGGAGGTGCGTCTGG | TTGAGGGTGGCGGGTTT |
| Ribosomal protein S20 | CCCCTGTTGAGGCTGAG | TCCACGGATAAGGTCTGC |

**Supplementary** **Table S2** Summary of the mapping of the transcription data from 10 pooled juvenile Atlantic salmon transcriptome samples to the Atlantic salmon (*Salmo salar*) draft genome.

| Sample name | Left/Input | Left/Mapped | Right/Input | Right/Mapped | Overall read mapping rate |
| --- | --- | --- | --- | --- | --- |
| LaAS1 | 15590578 | 83.60% | 15590578 | 78.90% | 81.30% |
| LaAS2 | 15580431 | 83.30% | 15580431 | 78.90% | 81.10% |
| LaCH | 16726316 | 83.20% | 16726316 | 79.00% | 81.10% |
| LaBT | 15832117 | 80.20% | 15832117 | 72.40% | 76.30% |
| LaRT | 15406518 | 77.60% | 15406518 | 68.20% | 72.90% |
| SeAS1 | 16253754 | 82.00% | 16253754 | 77.00% | 79.50% |
| SeAS2 | 16941555 | 82.70% | 16941555 | 78.10% | 80.40% |
| SeCH | 14921851 | 81.40% | 14921851 | 76.60% | 79.00% |
| SeBT | 16245883 | 80.00% | 16245883 | 75.30% | 77.70% |
| SeRT | 16431778 | 81.60% | 16431778 | 75.50% | 78.50% |

**Supplementary** **Table S3** Relative transcription of the 10 genes which showed a significant response to competition with all the three non-native salmonids for the LaHave Atlantic salmon (*Salmo salar*) population.

| Gene ID | Description | RPKM | | | |
| --- | --- | --- | --- | --- | --- |
|  |  | LaAS | LaBT | LaCH | LaRT |
| XLOC_006573 | mesoderm induction early response 2 isoform X2 | 1.06 | 6.96 | 5.56 | 11.75 |
| XLOC_007997 | NA | 1.80 | 6.35 | 0 | 9.36 |
| XLOC_026040 | PREDICTED: hypothetical protein LOC100636600 | 4.51 | 0 | 0 | 0 |
| XLOC_035303 | unnamed protein product | 1.01 | 18.66 | 9.30 | 30.76 |
| XLOC_038248 | reverse transcriptase | 0.75 | 0 | 2.88 | 4.79 |
| XLOC_043264 | fish virus induced TRIM | 1.54 | 5.54 | 6.05 | 8.12 |
| XLOC_044401 | NA | 3.32 | 0.04 | 0 | 0.08 |
| XLOC_045484 | NA | 2.09 | 11.99 | 13.40 | 9.74 |
| XLOC_051766 | unnamed protein product | 2.12 | 10.75 | 0 | 7.91 |
| XLOC_059159 | NA | 1.60 | 7.50 | 20.15 | 8.16 |

Note: LaAS represents LaHave Atlantic salmon reared alone; LaBT, LaCH, and LaRT represent LaHave Atlantic salmon reared with brown trout, Chinook salmon, and rainbow trout, respectively. NA indicates that the gene sequence did not match any sequences in the non-redundant (nr) database.

**Supplementary** **Table S4** Relative transcription of the nine genes which showed a significant response to competition with all the three non-native salmonids for the Sebago Atlantic salmon (*Salmo salar*) population.

| Gene ID | Description | RPKM | | | |
| --- | --- | --- | --- | --- | --- |
|  |  | SeAS | SeBT | SeCH | SeRT |
| XLOC_005337 | Somatostatin-1A precursor | 16.53 | 1.32 | 0.12 | 2.06 |
| XLOC_005338 | Somatostatin-2 precursor | 10.51 | 0.22 | 0.16 | 0.90 |
| XLOC_005514 | Somatostatin-1A precursor | 14.27 | 0.52 | 0.25 | 1.58 |
| XLOC_010401 | Dok-7-like isoform X1 | 1.09 | 4.65 | 0 | 4.98 |
| XLOC_013971 | phenylethanolamine N-methyltransferase-like | 6.35 | 9.04 | 10.99 | 0.05 |
| XLOC_025514 | NA | 11.93 | 0 | 0 | 0 |
| XLOC_026425 | glucagon-1 precursor | 8.76 | 1.18 | 0 | 0.47 |
| XLOC_031041 | Insulin precursor | 45.10 | 5.19 | 0.27 | 4.09 |
| XLOC_035700 | NA | 24.35 | 32.63 | 1.06 | 0.37 |

Note: SeAS represents Sebago Atlantic salmon reared alone; SeBT, SeCH, and SeRT represent Sebago Atlantic salmon reared with brown trout, Chinook salmon, and rainbow trout, respectively. NA indicates that the gene sequence did not match any sequences in the non-redundant (nr) database.

**Supplementary** **Table S5** Relative transcription of the 23 genes which showed a significant response to competition with Chinook salmon (*Oncorhynchus tshawytscha*) for both Atlantic salmon (*Salmo salar*) populations.

| gene ID | Description | RPKM | | | |
| --- | --- | --- | --- | --- | --- |
|  |  | LaAS | LaCH | SeAS | SeCH |
| XLOC_000035 | hypothetical protein EAI_17313 | 1.56 | 0 | 2.17 | 0.24 |
| XLOC_003299 | apolipo A-II precursor | 4.63 | 0.08 | 10.02 | 0.77 |
| XLOC_012227 | proglucagon II | 0.48 | 3.82 | 2.18 | 0 |
| XLOC_014649 | Apolipo A-I precursor | 1.23 | 0.03 | 1.97 | 0.16 |
| XLOC_015883 | AMBP precursor | 1.52 | 0.16 | 3.38 | 0.29 |
| XLOC_017724 | apolipo A-I precursor | 13.38 | 0.07 | 17.36 | 1.66 |
| XLOC_020727 | alpha-2-HS-glyco -like | 2.31 | 0 | 4.05 | 0.62 |
| XLOC_025691 | beta-2-glyco 1-like | 1.27 | 0 | 2.35 | 0.09 |
| XLOC_026300 | complement C5 | 1.63 | 0 | 2.38 | 0.15 |
| XLOC_028644 | Serpina1 , partial | 6.22 | 0 | 8.91 | 1.05 |
| XLOC_028948 | apolipo B-100-like | 0.99 | 0 | 2.61 | 0.22 |
| XLOC_029296 | fibrinogen gamma chain precursor | 2.79 | 0.03 | 4.95 | 0.58 |
| XLOC_029773 | collagen alpha-1 chain-like isoform X3 | 0.60 | 3.89 | 6.56 | 0.60 |
| XLOC_029784 | Serotransferrin-1 precursor | 3.54 | 0.46 | 7.73 | 1.49 |
| XLOC_035374 | Type-4 ice-structuring precursor | 0.83 | 0 | 1.38 | 0.11 |
| XLOC_038073 | NA | 1.11 | 4.55 | 2.86 | 9.81 |
| XLOC_038626 | apolipo A-II precursor | 5.17 | 0 | 10.72 | 1.21 |
| XLOC_039508 | warm temperature acclimation-related 65 kDa | 5.21 | 0.16 | 15.25 | 1.61 |
| XLOC_040523 | trout C-polysaccharide binding 1, isoform 1 | 4.11 | 0 | 9.37 | 0.58 |
| XLOC_040524 | trout C-polysaccharide binding 1, isoform 1 | 0.93 | 0 | 2.89 | 0.04 |
| XLOC_043208 | fibrinogen beta chain-like | 3.78 | 0 | 5.63 | 0.72 |
| XLOC_049651 | serum albumin 2 precursor | 7.45 | 0.29 | 14.16 | 2.39 |
| XLOC_058750 | NA | 1.19 | 8.56 | 9.80 | 1.74 |

Note: LaAS represents LaHave Atlantic salmon reared alone; LaCH represents LaHave Atlantic salmon reared with Chinook salmon; SeAS represents Sebago Atlantic salmon reared alone; and SeCH represents Sebago Atlantic salmon reared with Chinook salmon. NA indicates that the gene sequence did not match any sequences in the non-redundant (nr) database.

**Supplementary** **Table S6** Relative transcription of the 13 genes which showed response to competition with brown trout (*Salmo trutta*) for both Atlantic salmon (*Salmo salar*) populations.

| Gene ID | Description | RPKM | | | |
| --- | --- | --- | --- | --- | --- |
|  |  | LaAS | LaBT | SeAS | SeBT |
| XLOC_004403 | NA | 1.26 | 6.69 | 2.40 | 0.14 |
| XLOC_019565 | NA | 5.46 | 0 | 19.81 | 0 |
| XLOC_023427 | talin-1, partial | 4.02 | 0.76 | 1.58 | 6.24 |
| XLOC_034531 | dnaJ homolog subfamily B member 5-like | 1.31 | 0.18 | 0.08 | 0.68 |
| XLOC_038248 | reverse transcriptase | 0.75 | 0 | 2.19 | 7.59 |
| XLOC_039146 | unnamed protein product | 4.48 | 0.59 | 0.08 | 2.93 |
| XLOC_042761 | paternally-expressed gene 3 -like | 0.48 | 3.87 | 0.07 | 1.54 |
| XLOC_045381 | hypothetical protein CAPTEDRAFT_85835, partial | 2.30 | 0 | 0.76 | 3.92 |
| XLOC_047231 | hypothetical protein V500_07678 | 0.87 | 0 | 0.93 | 4.40 |
| XLOC_050042 | E3 ubiquitin- ligase HERC2 isoform X4 | 64.47 | 9.67 | 14.36 | 101.10 |
| XLOC_054691 | endonuclease domain-containing 1 -like | 2.23 | 8.82 | 2.59 | 0.30 |
| XLOC_059047 | NA | 0.30 | 9.31 | 15.46 | 1.88 |
| XLOC_059836 | NA | 7.66 | 38.60 | 5.07 | 30.75 |

Note: LaAS represents LaHave Atlantic salmon reared alone; LaBT represents LaHave Atlantic salmon reared with brown trout; SeAS represents Sebago Atlantic salmon reared alone; and SeBT represents Sebago Atlantic salmon reared with brown trout. NA indicates that the gene sequence did not match any sequences in the non-redundant (nr) database.

**Supplementary** **Table S7** Relative transcription of the 19 genes which showed response to competition with rainbow trout (*Oncorhynchus mykiss*) for both Atlantic salmon (*Salmo salar*) populations.

| Gene ID | Description | RPKM | | | |
| --- | --- | --- | --- | --- | --- |
|  |  | LaAS | LaRT | SeAS | SeRT |
| XLOC_002409 | NA | 14.78 | 1.17 | 0.41 | 6.85 |
| XLOC_002993 | NA | 1.03 | 5.74 | 1.89 | 0.11 |
| XLOC_004403 | NA | 1.26 | 9.28 | 2.40 | 0.20 |
| XLOC_005337 | Somatostatin-1A precursor | 10.55 | 1.44 | 16.53 | 2.06 |
| XLOC_005514 | Somatostatin-1A precursor | 6.87 | 0.52 | 14.27 | 1.58 |
| XLOC_010417 | transposase | 0.04 | 1.07 | 1.73 | 0.26 |
| XLOC_012848 | RNA-directed DNA polymerase from mobile element jockey-like, partial | 6.63 | 1.12 | 4.00 | 0 |
| XLOC_024287 | NA | 0.03 | 1.28 | 0.45 | 0 |
| XLOC_026425 | glucagon-1 precursor | 4.73 | 0.52 | 8.76 | 0.47 |
| XLOC_032189 | unnamed protein product | 0.54 | 2.33 | 1.25 | 0.22 |
| XLOC_034294 | tyrosine aminotransferase | 0.20 | 1.55 | 1.22 | 0 |
| XLOC_038248 | reverse transcriptase | 0.75 | 4.79 | 2.19 | 9.24 |
| XLOC_040524 | trout C-polysaccharide binding 1, isoform 1 | 0.93 | 4.25 | 2.89 | 0.44 |
| XLOC_042575 | NA | 1.13 | 8.75 | 3.89 | 0.45 |
| XLOC_042576 | NA | 1.25 | 15.87 | 3.18 | 0 |
| XLOC_048497 | hypothetical protein VOLCADRAFT_70901 | 2.51 | 0.14 | 2.27 | 0.13 |
| XLOC_051384 | fatty acid synthase-like | 3.06 | 0.40 | 0.44 | 2.55 |
| XLOC_058750 | NA | 1.19 | 8.81 | 9.80 | 0.15 |
| XLOC_060500 | hypothetical protein | 2.47 | 31.17 | 10.15 | 1.97 |

Note: LaAS represents LaHave Atlantic salmon reared alone; LaRT represents LaHave Atlantic salmon reared with rainbow trout; SeAS represents Sebago Atlantic salmon reared alone; and SeRT represents Sebago Atlantic salmon reared with rainbow trout. NA indicates that the gene sequence did not match any sequences in the non-redundant (nr) database.
